# Syrup of the Lacto-Phosphate of Lime

**Published:** 1872-10

**Authors:** 


### Syrup of the Lacto-Phosphate of Lime.

For this the *Chemist and Druggist* gives this formula:

| | |
|---|---|
| Concentrated lactic acid, | fluid dr. j. |
| Magma of freshly precipitated phosphate of lime, as much as will dissolve. | |
| Orange flower water, | fluid dr. j. ½ |
| Water up to. | " " |
| White sugar, | " 11 |

Mix the lactic acid with two ounces of water, and saturate it with the magma. Put the liquid upon a filter, and add the rest of the water until eight fluid ounces of filtrate are obtained. Pour this upon the sugar contained in a bottle; shake occasionally until solution is effected, and strain. No heat ought to be applied, else the syrup assumes a milky appearance. The syrup thus prepared contains between two and three grains of dry phosphate of lime in each fluid drachm, besides the lactic acid.

